# Impact of Sodium Nitrite Reduction on Lipid Oxidation and Antioxidant Properties of Cooked Meat Products

**DOI:** 10.3390/antiox9010009

**Published:** 2019-12-21

**Authors:** Małgorzata Karwowska, Anna Kononiuk, Karolina M. Wójciak

**Affiliations:** Department of Meat Technology and Food Quality, University of Life Sciences in Lublin, Skromna 8, 20-704 Lublin, Poland; anna.kononiuk@up.lublin.pl (A.K.); Karolina.wojciak@up.lublin.pl (K.M.W.)

**Keywords:** sodium nitrite, cooked meat products, lipid oxidation, hemeprotein

## Abstract

Oxidation processes are responsible for reduction of the sensory and nutritional quality of meat and meat products, thus affecting consumer acceptance. The use of sodium nitrite in meat processing is an important factor limiting these changes. Therefore, eliminating this substance from the recipe of meat products to increase their nutritional value is not an easy challenge. The aim of this study was to determine the effect of sodium nitrite reduction on the lipid oxidation (peroxide value, thiobarbituric acid reactive substances), and color parameters (CIE L*a*b*, total heme pigment and heme iron, nitrosylmyoglobin) in cooked meat products during 15 days of vacuum storage. The antioxidant properties of products and isolated peptides (2,2-azino-bis-3-ethylbenzothiazoline-6-sulfonic acid (ABTS•), 2,2-diphenyl-1-picrylhydrazyl (DPPH) radical scavenging activity, ferric-reducing antioxidant power) were also evaluated. Experimental material included four different sample groups of cooked meat products produced with various percentages of sodium nitrite (0, 50, 100, and 150 mg kg^−1^). It was shown that the sodium nitrite dose had no statistically significant effect on lightness (L*) and redness (a*) values, as well as nitrosylmyoglobin content. Along with decreasing the share of sodium nitrite in the samples, the thiobarbituric acid reactive substances (TBARS) value increased from 0.43 mg kg^−1^ for samples with 150 mg kg^−1^ at day 0 to 3.14 mg kg^−1^ for samples without nitrite at day 15. The total ABTS scavenging capacity of the cooked meat samples was in the range 2.48 to 4.31 eqv. mM Trolox per g of product throughout the entire storage period. During storage, the ferric-reducing antioxidant power of samples with nitrite increased from 0.25 to 0.38 eqv. mg/mL ascorbic acid per g of product. In conclusion, reduction of nitrite to the level of 50 mg kg^−1^ seemed to be comparable with the traditional use of nitrite in meat products in terms of the physicochemical properties and properties related to lipid oxidation, as well as total antioxidant capacity and peptide antioxidant capacity.

## 1. Introduction

Meat products are complex food matrices composed of various compounds (protein, lipids, fat-soluble vitamins, minerals, and bioactive compounds), which makes them very susceptible to chemical deterioration, mainly oxidation processes [1]. Oxidative processes reduce the shelf-life of the final product, leading to a loss of nutritional value, due to the degradation of fatty acids and vitamins, and affecting the organoleptic characteristics, e.g., color, texture, smell, and taste [2]. Moreover, lipid oxidation products have deleterious biological effects on the human body and are involved in several human pathologies, including atherosclerosis, cancer, inflammation, and aging processes [3,4].

Lipid oxidation in meat and meat products can be triggered by metal ions, either in the form of hemeproteins or in free form, which can donate electrons leading to increased rates of free radical production [5]. Therefore, meat and meat products with higher concentrations of myoglobin are more susceptible to lipid oxidation [6]. From hemeproteins, free iron can be released by the destruction of heme groups during cooking or storage [7]. As Amaral et al. [1] describe, the interaction of metmyoglobin with hydrogen peroxide or lipid peroxides causes the formation of ferrylmyoglobin, which can initiate free radical chain reactions. According to Byrne et. al. [8], cooked meat is more susceptible to lipid oxidation than raw meat because cooking temperature leads to the release of heme iron inducing production of free radicals. On the other hand, lipid oxidation increases the oxidation of hemeproteins. During lipid oxidation, a wide variety of aldehydes are produced that alter hemeprotein redox stability [1].

Curing is a conservation technique widely used to prolong the shelf-life of meat products due to the fact that nitrite retards lipid oxidation processes. Nitrite is a typical curing agent and acts against lipid oxidation through various mechanisms. One of these mechanisms is associated with binding of heme and prevention of the release of the catalytic iron. Nitrite also has the ability to bind heme and non-heme iron and inhibit catalysis. The third mechanism indicated in the literature consists in stabilization of lipids against oxidation [9]. On the other hand, many scientific papers have written about the negative effect of nitrite on human health: a high intake of nitrite presents a risk to consumer health due to the production of carcinogenic nitrosamines, and possible allergenic and vasodilator effects [10]. A link between the ingestion of N-nitroso compounds and the incidence of childhood leukemia, brain tumors, and colorectal cancer has also been suggested [11]. The formation of nitrosamines by reactions between a nitrosation agent and a secondary amine depends on many factors, including nitrite level, processing, decarboxylase activity of microorganisms, residual nitrite, acidity, and water activity [12,13]. Hence, public health concerns are nowadays related to the formation of carcinogenic nitrosamines rather than to the nitrite itself. However, limiting the use of nitrates in meat processing presents some difficulties. While the use of sodium chloride with nitrate during curing has many technological benefits, sodium chloride used alone has a pro-oxidant effect in meat products [14]. Min and Ahn [15] described several mechanisms by which NaCl promotes lipid oxidation. It is suggested that sodium chloride disrupts the structural integrity of membranes enabling catalysts to have access to substrates. Another explanation is that sodium chloride can increase the activity of ionic iron or decrease the activity of antioxidant enzymes (catalase, glutathione peroxidase, superoxide dismutase).

According to commission regulation (EU) No 1129/2011, the amount of nitrite permitted for use as a food additive in cured meat is currently 150 mg kg^−1^. Despite the difficulties arising from the reduction of sodium nitrate, some researchers indicate the possibility of reducing its addition to meat products. As reported by Rivera et al. [10], 20–50 ppm nitrite concentrations are required to retard rancidity. The results obtained by Wójciak et al. [14] revealed that sodium nitrite reduction to 100 mg kg^−1^ would be sufficient for minced roasted beef, without significant effects on color and oxidative stability.

Therefore, the aim of the present study is to determine the effect of sodium nitrite reduction on lipid oxidation and the color (CIEL*a*b*, total heme pigments, heme iron content, nitrosylmyoglobin) of cooked meat products during 15 days of vacuum storage. The antioxidant properties of products and isolated peptides are also evaluated.

## 2. Materials and Methods

### 2.1. Experimental Setup and Preparation of Cooked Meat Products

Four different sample groups of cooked meat products were produced with various percentages of sodium nitrite (0, 50, 100, and 150 mg kg^−1^). The sample groups were listed as: PP0—a cooked sausages sample without sodium nitrite addition; PP50—a cooked sausages sample with sodium nitrite addition in the amount of 50 mg kg^−1^; PP100—a cooked sausages sample with sodium nitrite addition in the amount of 100 mg kg^−1^; and PP150—a cooked sausages sample with sodium nitrite addition in the amount of 150 mg kg^−1^. Trimmed loins (*Musculus longissimus thoracis*) from Polish large white purebred fatteners with an average weight of 2.5 ± 0.5 kg were obtained from a local slaughterhouse near Biłgoraj (Poland) at 24 h postmortem. The loins were divided into four parts of about 500 g. The loins underwent salting (PP0) with sea salt (Cuor Di Mare, Italy) or curing (PP50, PP100, PP150) with a mixture of curing salt (containing sea salt and sodium nitrate). Salting/curing was carried out using a surface massage at 48 h postmortem. After completion of salting/curing, all of the loins were kept at 4 °C for 2 days to allow the salt or curing mixture to penetrate. Then, the meat was heat-treated in a cooking chamber at 85 °C to obtain 70 °C in the geometric center of the product. The products were cooled, vacuum-packed in nylon-polyethylene bags, and stored at 4 °C until analysis. All treatments were replicated independently twice. For each replicate, eight cooked products were produced per treatment. Samples at day 0 were taken for testing of proximate chemical composition, pH, water activity, color parameters, total pigments and heme iron content, total antioxidant capacity, and antioxidant properties of extracted peptides; whereas samples at 5, 10, and 15 days of storage were tested for pH, water activity, color parameters, total pigments and heme iron content, total antioxidant capacity, and antioxidant properties of extracted peptides.

### 2.2. Proximate Chemical Composition

The collagen, moisture, protein, and fat contents were determined using a Food Scan Lab 78810 (Foss Tecator Co., Ltd., Hillerod, Denmark). Briefly, approximately 200 g of a homogenized sample (each) was distributed in the instrument’s round sample dish and loaded into the instrument’s sample chamber. Each sample was analyzed three times.

### 2.3. pH Measurements

10 g of a minced sample was homogenized with 50 mL of de-ionized water for 1 min using a homogenizer (IKA T25, IKA^®^-Werke GmbH & CO. KG, Staufen, Germany). The pH was measured with a CPC-501 digital pH meter (Elmetron, Zabrze, Poland) equipped with a temperature sensor and pH electrode (ERH-111, Hydroment, Gliwice, Poland) calibrated with buffer solutions (pH 4.0, 7.0, 9.0). All determinations were performed in triplicate.

### 2.4. Water Activity Measurements

Water activity (a_w_) was measured at the temperature of 20 °C using a water activity analyzer (Novasina AG, Lachen, Switzerland), which gives temperature controlled measurements. The analyzer had been calibrated with Novasina SAL-T humidity standards (33%, 75%, 84%, and 90% relative humidity). All determinations were performed in triplicate.

### 2.5. Color Measurements

Color measurements were determined on the cross-section just after the product was cut. Hunter L*, a*, and b* values were measured using an X-Rite 8200 colorimeter (X-Rite, Inc., Michigan, USA) calibrated using the black glasses and white tiles provided. Samples were analyzed directly over the 8 mm aperture, with D65 illumination configurations, 10° viewing angle. Color coordinates were determined using the CIE Lab system. The results were expressed as lightness (L*), redness (a*), and yellowness (b*). Six determinations were carried out for each treatment, avoiding areas that had an excess of fat.

### 2.6. Total Pigments, Heme Iron, and Nitrosylmyoglobin Content

The total pigments, heme iron and nitrosylmyoglobin content were determined according to the procedure proposed by Hornsey [16] with a slight modification according to Karwowska and Dolatowski [17]. The amount of total pigments and heme iron content were calculated according to Lee et al. [18] and expressed in ppm. The amount of nitrosylmyoglobin was calculated by multiplying the absorbance at 540 nm by 290 [16]. All determinations were performed in triplicate.

### 2.7. Analysis of Lipid Oxidation

The extent of lipid oxidation in the product was assessed by measuring the amount of thiobarbituric acid reactive substances (TBA) according to the procedure described by Pikul et al. [19]. The values are expressed as mg of malondialdehyde (MDA) per kilogram of sample. The determinations were made in triplicate.

The peroxide value (POV) was determined based on the method described by Koniecko [20]. POV was calculated by the following equation and expressed as milliequivalent peroxide per kilogram of sample:

POV (meq O_2_ kg^−1^) = [(S × N)/W] × 100 (1)

where “S” is the volume of titration (mL), “N” is the normality of sodium thiosulfate solution (N = 0.01) and “W” is the sample weight (g).

### 2.8. Total Antioxidant Capacity (TAC)

Extracts for Total Antioxidant Capacity (TAC) measurement were prepared according to method of Korzeniowska et al. [21]. Briefly, 5 g of samples were homogenized on ice with water, after one-hour extraction, the homogenate was centrifuged at 5000× *g* for 30 min at 4 °C. TAC was measured in obtained extracts using 2-azino-bis-3-ethylbenzothiazoline-6-sulfonic acid (ABTS•^+^) radical scavenging activity and ferric-reducing antioxidant power (RP, reducing power). The methodology for determining ABTS•^+^ radical scavenging activity and RP (reducing power) is presented in Section 2.9. All analyses were performed in triplicate.

### 2.9. Antioxidant Properties of Peptides

#### 2.9.1. Extraction of Peptides

Extraction of peptides was performed according to the method of Zhu et al. [22] with slight modifications. Then, 2.5 g of samples were homogenized with 10 mL of 0.01 M HCl for 1 min with cooling on ice using a homogenizer (IKA T25, Staufen, Germany). The homogenate was centrifuged at 5000× *g* for 30 min at 4 °C, and after filtration through glass wool, 5 mL of supernatant was added to 15 mL of frozen ethanol. The mixture was kept at 4 °C overnight and then centrifuged at 5000× *g* for 30 min at 4 °C. The supernatant was collected and stored at −20 °C in an evaporator until concentrated. The concentrated extract was dissolved in 0.01 M HCl and filtered through a 0.45 μm nylon membrane filter (AlfaChem, Toruń, Poland) and stored at −20 °C prior to use. Antioxidant activity of peptides was measured using ABTS and DPPH radical scavenging activity, as well as ferric-reducing antioxidant power (reducing power).

#### 2.9.2. ABTS•^+^ (2-Azino-bis-3-ethylbenzothiazoline-6-sulfonic Acid) Radical Scavenging Activity

ABTS•^+^ radical scavenging activity was measured using the ABTS radical cation decolorization assay described by Re et al. [23]. The ability of the peptides to scavenge the ABTS•^+^ radicals was evaluated with reference to the Trolox standard curve (0–5 mg 100 mL^−1^). The absorbance at 734 nm was read using a UV–visible spectrophotometer (Nicolet Evolution 300, Thermo Electron Corp., Waltham, MA, USA).

Results were shown as the ability of the extract as well as the peptides to scavenge ABTS radical cations and expressed as Trolox equivalent mM per g of product.

#### 2.9.3. DPPH (2,2-Diphenyl-1-picrylhydrazyl) Radical Scavenging Activity

DPPH radical scavenging activity of peptides isolated from products was assessed according to the method described by Zhu et al. [22]. The ability of extracts and peptides to scavenge DPPH free radicals was evaluated with reference to the Trolox standard curve (0–5 mg 100 mL^−1^). The absorbance at 517 nm was measure using a UV-visible spectrophotometer (Nicolet Evolution 300, Thermo Electron Corp., Waltham, MA, USA). The results were shown as the ability of peptides to scavenge DPPH free radicals and expressed as Trolox equivalent mM per g of product.

#### 2.9.4. Ferric-Reducing Antioxidant Power (RP—Reducing Power)

The ability of meat–water extracts, as well as peptides to reduce iron from the Fe^3+^ (ferric) oxidation state to the Fe^2+^ (ferrous) oxidation state, was determined by the method described by Mora et al. [24]. The results were calculated with reference to the results obtained for the ascorbic acid standard curve (0–0.15 mg 100 mL^−1^). The absorbance at 700 nm was read using a UV–visible spectrophotometer (Nicolet Evolution 300, Thermo Electron Corp., Waltham, MA, USA). The results were shown as the ability of extracts and peptides to reduce iron from the Fe^3+^ to the Fe^2+^ oxidation state and expressed as equivalent mg ascorbic acid per g of product.

### 2.10. Statistical Analysis

The collected data were analyzed using Statistica version 13.3 software (Dell Inc., Round Rock, TX, USA) and expressed as mean ± standard deviations. Effects between categorical factors (day and variant) and variables between subgroups were analyzed using factorial ANOVA. Homogeneity of variances was checked by Levene’s test. Post hoc comparison was specified based on Tukey’s test. All differences were significant at *p* ≤ 0.05. To evaluate and classify the main variables of all samples, principal component analysis (PCA) was applied. The number of principal components was determined based on the percentages of variance explained, Kaiser’s criterion, and the Cattell test.

## 3. Results and Discussion

The proximate chemical composition of four formulations is shown in Table 1. As expected, there were no significant differences between samples in terms of moisture, protein, fat, collagen, and salt contents.

Table 2 shows the statistical significance (*p*-values) of the main factors and their interactions on the measured parameters. Variant was a significant factor (0.001 < *p* < 0.01) for all parameters except nitrosylmyoglobin content. Day of storage (0, 5, 10, 15) was a highly significant factor (*p* < 0.001) for almost all parameters except lightness (L*), yellowness (b*), nitrosylmyoglobin, and pH, where no statistical significance of this factor was observed. TBARS, as well as antioxidant activity (TAC_ABTS, TAC_RP, PEP_ABTS, PEP_RP, PEP_DPPH) of samples, were highly significantly (*p* < 0.001), determined by day of processing, variant, and interaction between them.

Table 3 shows pH and water activity values of produced cooked meat products with varying levels of nitrite. At the beginning of the experiment (day 0), lower pH values were detected for the variants containing nitrite (PP50, PP100, PP150) compared to the uncured variant (PP0). Nitrite concentration did not alter the acidity of the cooked products. Similar results have been achieved by other researchers [25,26]. The acidity of almost all samples did not change during storage, except for sample PP150. A significantly lower pH was observed in the sample, with the highest concentration of nitrite during storage. During the entire storage period, the samples with sodium nitrite were characterized by lower pH values compared to uncured samples; therefore, they were less susceptible to microbial spoilage [27]. It was also observed that on day 15, the pH of the sample with reduction of sodium nitrite to 50 mg kg^−1^ did not differ significantly from the sample with the traditional use of nitrite (PP150). Elimination of nitrite (PP0) resulted in a significantly higher pH of the cooked meat product compared to the samples with nitrite.

In terms of water activity, it was observed that sodium nitrite inclusion level and storage time were significant factors. As stated by Honikel [27], water activity is a microbiological hurdle and may prolong the shelf-life of meat products. At the beginning of the experiment (0, 5 day), no differences were observed for a_w_ between the meat products samples (Table 3). A decrease in the water activity of tested samples was observed on day 15. A significantly lower water activity was observed in sample PP150 compared to other samples on days 10 and 15. In contrast to our findings, Wójciak et al. [14] observed a gradual increase in the water activity of roasted beef samples with different levels of sodium nitrite. As shown in Table 4, moderate significant (*r* > 0.72) correlations were found between water activity and the antioxidant properties of peptides (PEP_ABTS, PEP_RP, PEP_DPPH).

The color of meat products is noted to be an essential parameter and considered a key purchase criterion by consumers [28]. It depends on a combination of different factors, including fat and moisture content; however, the most important is the chemical form and concentration of myoglobin. Hunter L*a*b* values, total heme pigment, heme iron, and nitrosylmyoglobin of meat products samples are shown in Table 5 and Table 6. It can be seen that sodium nitrite dose had no statistically significant effect on lightness (L*) and redness (a*) values or on nitrosylmyoglobin content. Similarly, the results presented by Wójciak et al. [14] showed no effect from inclusion of different amounts of sodium nitrite on the redness of roasted beef. As shown in Table 5, the nitrite level had statistically significant effects on the total heme pigments and heme iron content. Initial values of total pigments and heme iron content on day 0 demonstrate that the PP50 sample was comparable with PP150. In contrast, at the end of experiment PP0 (uncured) showed significantly higher total pigments and heme iron content than those observed for PP150. Different results were obtained by Wójciak et al. [14], who showed that cured samples were characterized by the highest total pigment and heme iron content.

Lipid oxidation of cooked meat products with various doses of nitrite was monitored by measuring peroxide values (this measures the formation of peroxide or hydroperoxide groups that are the primary products of lipid oxidation) and TBA-reactive substances as markers for secondary products of lipid oxidation (Table 7). On day 0 (after production), POV values of cooked meat products were not significantly different between samples and amounted to 0.31 meq O_2_ kg^−1^ while in relation to TBARS values significant differences between samples were noted. As expected, the lowest content of secondary fat oxidation products reacting with thiobarbituric acid, both on day 0 and throughout the entire storage period, was characterized by the sample with the highest content of sodium nitrite (PP150). Along with a decrease in the share of sodium nitrite in the samples, the TBARS value increased. Antioxidant properties and mechanisms of sodium nitrite interaction are widely described in the literature [9,26]. Moawad et al. [26] also stated that, as the concentrations of nitrite increased in the formula, the TBARS of raw-cured sausages decreased. The main mechanism of nitrite antioxidant activity is related with nitric oxide, which can trigger lipid oxidation by chelating free radicals. Nitric oxide reacts with other radicals (hydroxyl radical, alkoxy radicals, and peroxyl radicals) interrupting radical chain reactions. NO can also bind to transitional metals, and this aspect is very important because the binding to ferrous heme complex decreases its probability of producing hydroxyl radicals in the Fenton reaction. As stated by Honikel [27], nitrite acts against lipid oxidation mainly due to oxygen deletion. The nitric oxide, formed from nitrite, can be oxidized to form NO_2_, causing oxygen sequestering. In such conditions, the oxidation of meat lipids is inhibited. Our data showed that nitrite reduction to 100 mg kg^−1^ did not change the TBARS value compared to the sample with the traditional use of nitrite (PP150). The reduction of sodium nitrite to 50 mg kg^−1^ caused an increase in TBARS by 0.9 mg kg^−1^ compared to the PP150 sample on days 15, however, it was 0.7 mg kg^−1^ lower compared to the sample without nitrite (PP0). These results confirmed the findings of Wójciak et al. [11], who reported that nitrite reduction to 100 mg kg^−1^ in minced roasted beef would be sufficient for maintaining oxidative stability. Sebranek and Bacus [28] reported that nitrite is effective at relatively low concentration.

As shown in Table 4, moderate significant (*r* > 0.53) correlations were found between water activity and POV and with TBARS (inverse). Lipid peroxides measured by POV are the primary products of oxidation and that is why an increase in hydroperoxides is often observed during the early stages of oxidation. These compounds are unstable, and as a result of decomposition, their content decreases in more advanced stages of oxidation [29]. In the present study, POV reduction and an increase in TBARS with storage time was observed.

Due to the multifunctional character of antioxidants present in meat and meat products, three radical scavenging capacity assays were applied to investigate heterogeneous samples since each assay involves different chemical mechanisms and may reflect different aspects of their antioxidant properties. Scavenging of DPPH radical allows evaluation of the hydrogen-donating potency of antioxidative compounds; the ABTS radical determines the single electron-transfer capabilities, while the ferric-reducing antioxidant power assay is an important indicator to estimate the antioxidant potential based on the chelating capacity of ferrous ion (Fe^3+^ to Fe^2+^) [30,31]. TAC (total antioxidant capacity) of the samples determined by the ABTS and ferric-reducing antioxidant power are presented in Table 8. The total ABTS scavenging capacity of the cooked meat samples was in the range 2.48 ± 0.17 eqv. mM Trolox per g of product to 4.31 ± 0.31 eqv. mM Trolox per g of product throughout the entire storage period. Comparing the ABTS and RP results of the meat products samples with and without nitrite, no significant differences were observed between samples at day 0. During storage, the ferric-reducing antioxidant power of samples with nitrite increased. The highest RP was observed in the case of sample PP150 after 10 days of storage. At the end of experiment (days 15), cooked meat product with 50 mg kg^−1^ addition of nitrite was characterized by a similar TAC (ABTS, RP) compared to the sample with the traditional use of nitrite (PP150). The observed effect of nitrite on TAC could be associated with the ability of nitrite to bind heme and non-heme iron [31].

Different components are responsible for this TAC. It has been noted that proteins and peptides exert an important antioxidant action in meats and meat products due to their ability to scavenge free radicals and chelate prooxidative metals [32]. Compounds that result from protein degradation may be responsible for the antioxidant properties of meat products. The degradation of muscle proteins during the manufacturing process and storage results in the formation of small peptides. The consequences of the antioxidant properties of the peptides isolated from experimental cooked meat products highlight significant differences between the samples (Table 9). At the beginning of the experiment, antioxidant activity against the ABTS• radical with the various doses of nitrite was not significantly different between the samples. However, a decrease in this capacity was observed during chilling storage. Similar relationships were observed when antioxidant activity was determined by DPPH radical scavenging activity and ferric-reducing antioxidant power. On day 15, cooked meat product samples with sodium nitrite in the amounts of 100 and 150 mg kg^−1^ (PP100, PP150) were characterized by significantly higher antioxidant activity against the ABTS• radical compared to the sample without sodium nitrite (PP0), while no statistically significant differences in DPPH were found between samples on this storage day. In terms of ferric-reducing antioxidant power at the end of storage (day 15), the highest RP among the samples was characterized by the PP100 variant. However, reduction of nitrite to 50 mg kg^−1^ allowed for obtaining the same RP value compared to the sample with sodium nitrite addition in the amount of 150 mg kg^−1^. This confirms that antioxidant properties of nitrite is associated with the ability of nitrite to bind heme and non-heme iron. Despite the antioxidant properties of nitrite, no clear increase in activity was observed with increasing nitrite content in the samples. Wójciak et al. [14] obtained similar results for uncured roast beef. They suggested that specific protein breakdown products are involved in the action against free radicals in meat. As presented by Sarmadi et. al. [33], the antioxidant potential of peptides is related to their metal ion chelation, lipid peroxidation inhibition, and radical scavenging properties. It has also been indicated that the structure of peptides with its amino acid sequence can influence their antioxidative potential [33]. The statistical analysis carried out in this study showed a positive correlation between PEP_DPPH and PEP_ABTS as well as between PEP_DPPH and PEP_RP (Table 9). Antioxidant peptides obtained from animal sources can exert not only nutritional value, but also bioavailability to benefit human health [34].

**Figure 1:**
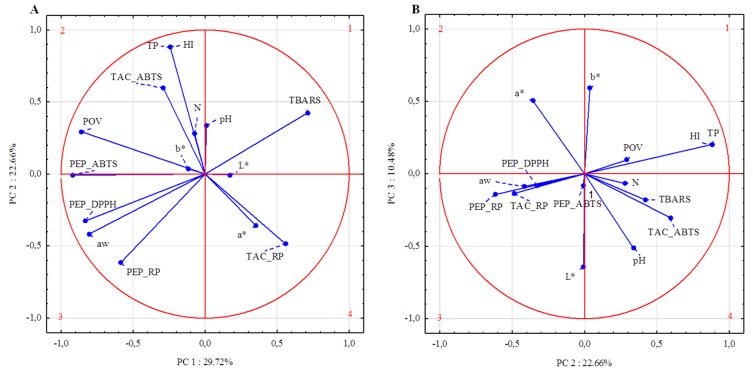
Loadings for: (**A**) the first two principal components; (**B)** second and third principal components. L*, a*, b*—color coordinates, N—nitrosylmyoglobin content, TP—total pigments content, HI—heme iron content, TAC_ABTS—ABTS•^+^ radical scavenging activity of meat—water extract, TAC_RP—reducing power on meat—water extract, PEP_ABTS—ABTS•^+^ radical scavenging activity of extracted peptides, PEP_DPPH—DPPH radical scavenging activity of extracted peptides; PEP_RP—reducing power of extracted peptides.

To provide a partial visualization of collected data dependences, PCA was performed. According to the results of total variance analyses, using the test proposed by Cattell and Kaiser’s criterion, the number of principal components was established (data not shown). The first three principal components explained 62.86% of the cumulative variance. The variables that most correlated to PC1 included TAC_RP, all antioxidant activity assays (ABTS^+^, DPPH, RP) for peptides, primary (POV) and secondary products of lipid peroxidation, as well as water activity. In contrast to the other variables, only TBARS, TAC_RP, L*, and a* were positively correlated to PC1 (Figure 1).

The highest correlations between variables allocated to the first component were observed between a_w_ and PEP_DPPH (correlation coefficient 0.86), PEP_RP (correlation coefficient 0.75), as well as PEP_ABTS (correlation coefficient 0.72) (Figure 1). In addition, PEP_ABTS is highly correlated with PEP_DPPH (correlation coefficient 0.77) and POV (correlation coefficient 0.75). The second principal component (explaining 22.66% of cumulative variance) included total pigments content, heme iron content, TAC_ABTS, and PEP_RP. Changes in TAC_ABTS were positively correlated with changes in TP and HI (correlation coefficient 0.57). There were no observed correlations between POV and other variables allocated to PC2. Other variables such as color coordinates (L*, a*, b*) and pH were most correlated with PC3. Nevertheless, no significant correlation between these parameters was observed (Table 10).

## 4. Conclusions

The reduction of nitrite in meat processing is beneficial to the health of the consumer; however, it is associated with many difficulties that result from the multidirectional impact of this additive in the meat product system. The results of the present study indicate that there is a possibility of nitrite reduction in cooked meat products. Reduction of nitrite to the level of 50 mg kg^−1^ seems to be comparable with the traditional use of nitrite in meat products (150 mg kg^−1^) in terms of the physicochemical properties and properties related to lipid oxidation. Moreover, meat products with 50 mg kg^−1^ addition of nitrite are characterized by a similar TAC, as well as peptide antioxidant capacity. Further research is needed to determine the effect of nitrite reduction using a combination of potential alternative compounds.

## Figures and Tables

**Table 1:** Proximate chemical composition of cooked meat product.

Component	Variant			
	PP0	PP50	PP100	PP150
Fat	4.44 ± 3.12	5.85 ± 2.06	4.76 ± 1.27	5.23 ± 0.60
Protein	31.85 ± 6.19	28.38 ± 0.28	28.68 ± 0.33	28.18 ± 2.06
Moisture	60.16 ± 11.24	64.31 ± 1.19	65.02 ± 0.69	64.84 ± 2.26
Collagen	0.37 ± 0.41	0.91 ± 0.26	0.75 ± 0.01	0.93 ± 0.18
Salt	2.80 ± 1.29	1.60 ± 0.18	1.74 ± 0.10	1.81 ± 0.11

No significant difference between samples. PP0—without sodium nitrite addition; PP50—with sodium nitrite addition in the amount of 50 mg kg^−1^; PP100—with sodium nitrite addition in the amount of 100 mg kg^−1^; PP150—with sodium nitrite addition in the amount of 150 mg kg^−1^.

**Table 2:** Statistical significance of main factors and their interactions with measured parameters.

Parameter	Variant	Day	Variant × Day
L*	***	n.s.	n.s.
a*	***	***	n.s.
b*	***	n.s.	n.s.
Nitrosylmyoglobin	n.s.	n.s.	**
Total pigments content	**	***	***
Heme iron content	**	***	***
a_w_	*	***	***
pH	***	n.s.	***
TBARS	***	***	***
POV	*	***	*
TAC_ABTS	***	***	***
TAC_RP	***	***	***
PEP_ABTS	***	***	***
PEP_DPPH	***	***	***
PEP_RP	***	***	***

Fixed effects: n.s.—no statistical significance (*p* > 0.05); *—*p* ≤ 0.05; **—*p* ≤ 0.01; ***—*p* ≤ 0.001. L*, a*, b*—color coordinates, TAC_ABTS—ABTS•^+^ radical scavenging activity of meat—water extract, TAC_RP—reducing power on meat—water extract, PEP_ABTS—ABTS•^+^ radical scavenging activity of extracted peptides, PEP_DPPH—DPPH radical scavenging activity of extracted peptides; PEP_RP—reducing power of extracted peptides.

**Table 3:** Physicochemical parameters of cooked meat products.

Parameter	Day	Variant			
		PP0	PP50	PP100	PP150
a_w_	0	0.977 ± 0.001 ^ab^	0.978 ± 0.000 ^ab^	0.978 ± 0.000 ^a^	0.977 ± 0.001 ^ab^
	5	0.976 ± 0.001 ^bc^	0.976 ± 0.001 ^bc^	0.977 ± 0.001 ^ab^	0.978 ± 0.001 ^ab^
	10	0.977 ± 0.001 ^ab^	0.977 ± 0.001 ^ab^	0.977 ± 0.000 ^abc^	0.975 ± 0.000 ^c^
	15	0.969 ± 0.001 ^d^	0.968 ± 0.001 ^d^	0.968 ± 0.001 ^de^	0.967 ± 0.000 ^e^
pH	0	5.80 ± 0.02 ^abc^	5.71 ± 0.02 ^de^	5.70 ± 0.01 ^de^	5.72 ± 0.10 ^bcd^
	5	5.80 ± 0.01 ^ab^	5.71 ± 0.02 ^cde^	5.69 ± 0.01 ^def^	5.61 ± 0.01 ^f^
	10	5.79 ± 0.04 ^bc^	5.74 ± 0.04 ^bcd^	5.72 ± 0.01 ^bcd^	5.63 ± 0.01 ^ef^
	15	5.88 ± 0.05 ^a^	5.70 ± 0.09 ^de^	5.71 ± 0.02 ^de^	5.63 ± 0.05 ^ef^

PP0—without sodium nitrite addition; PP50—with sodium nitrite addition in the amount of 50 mg kg^−1^; PP100—with sodium nitrite addition in the amount of 100 mg kg^−1^; PP150—with sodium nitrite addition in the amount of 150 mg kg^−1^. Means with different lowercase letters (a–f) differ significantly (*p* < 0.05).

**Table 4:** Correlation matrix.

	TAC_ABTS	PEP_ABTS	TAC_RP	PEP_RP	PEP_DPPH	TBARS	POV	L*	a*	b*	Nitrozylo-Myoglobin	TP	Haem Iron	aw	pH
TAC_ABTS	1.000														
PEP_ABTS	0.344	1.000													
TAC_RP	−0.134	−0.400	1.000												
PEP_RP	−0.010	0.554	0.170	1.000											
PEP_DPPH	0.026	0.773	−0.308	0.635	1.000										
TBARS	0.196	−0.601	0.337	−0.626	−0.575	1.000									
POV	0.391	0.753	−0.635	0.252	0.591	−0.492	1.000								
L*	0.093	−0.103	0.168	−0.034	−0.114	0.192	−0.118	1.000							
a*	−0.275	−0.292	0.497	0.030	−0.111	0.169	−0.359	−0.243	1.000						
b*	−0.179	0.048	-0.249	−0.135	0.119	−0.038	0.188	−0.106	0.157	1.000					
Nitrosylomyoglobin	0.147	0.001	-0.097	−0.010	0.004	0.054	0.074	−0.117	−0.079	−0.088	1.000				
Total pigments content	0.573	0.224	-0.452	−0.320	−0.116	0.168	0.448	−0.131	−0.205	0.079	0.230	1.000			
Heme iron	0.573	0.224	-0.452	−0.320	−0.116	0.168	0.448	−0.131	−0.205	0.079	0.230	1.000	1.000		
aw	0.064	0.724	-0.155	0.748	0.858	−0.609	0.526	−0.098	−0.060	0.092	−0.016	−0.158	-0.158	1.000	-
pH	0.151	0.020	-0.270	−0.268	0.105	0.380	−0.016	0.139	−0.256	−0.085	0.162	0.095	0.095	-0.005	1.000

Correlations higher than 0.500 and lower than −0.500 have been marked in red.

**Table 5:** L*a*b* color parameters of cooked meat products.

Parameter	Day	Variant			
		PP0	PP50	PP100	PP150
L*	0	77.55 ± 2.16 ^ab^	73.30 ± 1.76 ^b^	75.35 ± 2.77 ^ab^	76.22 ± 1.85 ^ab^
	5	78.14 ± 1.72 ^a^	76.21 ± 2.14 ^ab^	75.27 ± 3.76 ^ab^	77.81 ± 1.36 ^a^
	10	77.68 ± 1.47 ^a^	75.71 ± 1.93 ^ab^	77.36 ± 0.85 ^ab^	75.68 ± 2.98 ^ab^
	15	77.22 ± 1.18 ^ab^	76.73 ± 1.51 ^ab^	76.00 ± 1.63 ^ab^	77.53 ± 2.81 ^ab^
a*	0	2.44 ± 0.47 ^d^	3.32 ± 0.45 ^bcd^	2.75 ± 0.89 ^cd^	3.61 ± 0.34 ^abcd^
	5	3.51 ± 0.27 ^bcd^	4.46 ± 0.45 ^ab^	3.86 ± 0.52 ^abc^	3.71 ± 0.41 ^abcd^
	10	3.90 ± 0.29 ^abc^	4.89 ± 1.01 ^a^	4.43 ± 0.54 ^ab^	3.92 ± 0.31 ^abc^
	15	3.20 ± 1.08 ^bcd^	4.23 ± 0.77 ^ab^	3.64 ± 1.08 ^abcd^	3.96 ± 0.62 ^abc^
b*	0	12.62 ± 0.68 ^abc^	13.11 ± 0.44 ^a^	12.46 ± 0.25 ^abc^	12.82 ± 1.16 ^ab^
	5	12.77 ± 0.30 ^ab^	13.13 ± 0.72 ^a^	11.43 ± 0.78 ^c^	12.18 ± 0.33 ^abc^
	10	12.33 ± 0.57 ^abc^	13.11 ± 0.33 ^a^	11.90 ± 0.46 ^bc^	12.46 ± 0.41 ^abc^
	15	12.49 ± 0.31 ^abc^	12.27 ± 0.60 ^abc^	11.98 ± 0.69 ^abc^	12.61 ± 0.66 ^abc^

PP0—without sodium nitrite addition; PP50—with sodium nitrite addition in the amount of 50 mg kg^−1^; PP100—with sodium nitrite addition in the amount of 100 mg kg^−1^; PP150—with sodium nitrite addition in the amount of 150 mg kg^−1^. Means with different lowercase letters (a–f) differ significantly (*p* < 0.05).

**Table 6:** Total pigments, heme pigments, and nitrosylmyoglobin content in cooked meat products.

Parameter	Day	Variant			
		PP0	PP50	PP100	PP150
Nitrosylmyoglobins (ppm)	0	13.39 ± 0.43 ^ab^	14.07 ± 0.63 ^ab^	13.78 ± 0.30 ^ab^	13.92 ± 0.55 ^ab^
	5	13.44 ± 0.70 ^ab^	13.73 ± 0.44 ^ab^	13.87 ± 0.29 ^ab^	13.58 ± 0.67 ^ab^
	10	13.68 ± 0.22 ^ab^	13.58 ± 0.43 ^ab^	13.87 ± 0.34 ^ab^	13.49 ± 0.63 ^ab^
	15	14.36 ± 0.40 ^a^	13.24 ± 0.30 ^b^	14.07 ± 0.65 ^ab^	13.44 ± 0.30 ^ab^
Total pigments content (ppm)	0	58.25 ± 1.53 ^de^	70.27 ± 9.50 ^ab^	58.25 ± 2.50 ^de^	70.61 ± 2.90 ^a^
	5	64.71 ± 5.36 ^abcd^	63.58± 2.50 ^abcde^	62.90 ± 2.14 ^bcde^	61.77 ± 1.90 ^cde^
	10	46.92 ± 0.74 ^f^	48.28 ± 2.72 ^f^	48.17 ± 0.51 ^f^	47.60 ± 0.61 ^f^
	15	67.77 ± 3.86 ^abc^	63.58 ± 2.81 ^abcde^	61.77 ± 5.22 ^cde^	57.01 ± 1.79 ^e^
Heme iron content (ppm)	0	5.14 ± 0.14 ^de^	6.20 ± 0.84 ^ab^	5.14 ± 0.22 ^de^	6.23 ± 0.26 ^a^
	5	5.71 ± 0.47 ^abcd^	5.61 ± 0.22 ^abcde^	5.55 ± 0.19 ^bcde^	5.45 ± 0.17 ^cde^
	10	4.14 ± 0.07 ^f^	4.26 ± 0.24 ^f^	4.25 ± 0.05 ^f^	4.20± 0.05 ^f^
	15	5.98 ± 0.34 ^abc^	5.61 ± 0.25 ^abcde^	5.45 ± 0.46 ^cde^	5.03± 0.16 ^e^

PP0—without sodium nitrite addition; PP50—with sodium nitrite addition in the amount of 50 mg kg^−1^; PP100—with sodium nitrite addition in the amount of 100 mg kg^−1^; PP150—with sodium nitrite addition in the amount of 150 mg kg^−1^. Means with different lowercase letters (a–f) differ significantly (*p* < 0.05).

**Table 7:** TBARS and POV values in cooked meat products.

Parameter	Day	Variant			
		PP0	PP50	PP100	PP150
TBARS (mg kg^-1^)	0	0.88 ± 0.15 ^gh^	0.84 ± 0.22 ^ghi^	0.64 ± 0.09 ^hi^	0.43 ± 0.05 ^i^
	5	2.02 ± 0.06 ^c^	2.13 ± 0.12 ^bc^	1.42 ± 0.34 ^def^	1.17 ± 0.01 ^efg^
	10	1.45 ± 0.04 ^def^	1.47 ± 0.10 ^def^	1.24 ± 0.08 ^efg^	1.07 ± 0.03 ^fgh^
	15	3.14 ± 0.12 ^a^	2.47 ± 0.64 ^b^	1.75 ± 0.28 ^cd^	1.57 ± 0.10 ^de^
POV (meq O_2_ kg^-1^)	0	0.28 ± 0.04 ^a^	0.31 ± 0.02 ^a^	0.30 ± 0.06 ^a^	0.30 ± 0.03 ^a^
	5	0.21 ± 0.02 ^bcd^	0.21 ± 0.07 ^bcd^	0.21 ± 0.04 ^bc^	0.25 ± 0.06 ^ab^
	10	0.15 ± 0.01 ^cdef^	0.16 ± 0.01 ^cde^	0.08 ± 0.01 ^f^	0.11 ± 0.02 ^ef^
	15	0.13 ± 0.01 ^ef^	0.13 ± 0.01 ^ef^	0.10 ± 0.01 ^ef^	0.14 ± 0.01 ^def^

PP0—without sodium nitrite addition; PP50—with sodium nitrite addition in the amount of 50 mg kg^−1^; PP100—with sodium nitrite addition in the amount of 100 mg kg^−1^; PP150—with sodium nitrite addition in the amount of 150 mg kg^−1^. Means with different lowercase letters (a–i) differ significantly (*p* < 0.05).

**Table 8:** Total antioxidant capacity of cooked meat products.

Parameter	Day	Variant			
		PP0	PP50	PP100	PP150
ABTS (eqv. mM Trolox per g of product)	0	3.48 ± 0.24 ^bcd^	3.47 ± 0.20 ^bcd^	3.56 ± 0.16 ^bc^	3.29 ± 0.04 ^cdef^
	5	4.09 ± 0.22 ^a^	3.55 ± 0.37 ^bc^	4.31 ± 0.31 ^a^	4.12 ± 0.19 ^a^
	10	2.80 ± 0.14 ^fgh^	2.48 ± 0.17 ^h^	2.93 ± 0.19 ^efgh^	2.66 ± 0.69 ^gh^
	15	3.97 ± 0.12 ^ab^	3.18 ± 0.17 ^cdefg^	3.42 ± 0.09 ^cde^	2.96 ± 0.08 ^defgh^
RP (eqv. mg/mL ascorbic acid per g of product)	0	0.27 ± 0.01 ^fg^	0.25 ± 0.01 ^g^	0.25 ± 0.01 ^g^	0.25 ± 0.01 ^g^
	5	0.33 ± 0.01 ^de^	0.36 ± 0.01 ^ab^	0.35 ± 0.01 ^bcd^	0.35 ± 0.00 ^abc^
	10	0.34 ± 0.01 ^bcd^	0.35 ± 0.02 ^bcd^	0.36 ± 0.01 ^ab^	0.38 ± 0.01 ^a^
	15	0.29 ± 0.00 ^f^	0.33 ± 0.01 ^cde^	0.37 ± 0.00 ^a^	0.32 ± 0.02 ^e^

PP0—without sodium nitrite addition; PP50—with sodium nitrite addition in the amount of 50 mg kg^−1^; PP100—with sodium nitrite addition in the amount of 100 mg kg^−1^; PP150—with sodium nitrite addition in the amount of 150 mg kg^−1^. Means with different lowercase letters (a–h) differ significantly (*p* < 0.05).

**Table 9:** Peptide antioxidant capacity in cooked meat products.

Parameter	Day	Variant			
		PP0	PP50	PP100	PP150
ABTS (eqv. mM Trolox per g of product)	0	0.20 ± 0.01 ^a^	0.20 ± 0.01 ^a^	0.18 ± 0.01 ^a^	0.20 ± 0.01 ^a^
	5	0.20 ± 0.06 ^a^	0.13 ± 0.02 ^bc^	0.18 ± 0.01 ^a^	0.14 ± 0.01 ^b^
	10	0.14 ± 0.02 ^b^	0.10 ± 0.01 ^cd^	0.13 ± 0.01 ^bc^	0.10 ± 0.00 ^cd^
	15	0.05 ± 0.00 ^e^	0.07 ± 0.01 ^de^	0.08 ± 0.00 ^d^	0.09 ± 0.01 ^d^
DPPH (eqv. mM Trolox per g of product)	0	0.11 ± 0.01 ^a^	0.11 ± 0.01 ^ab^	0.09 ± 0.02 ^bcd^	0.09 ± 0.00 ^cd^
	5	0.08 ± 0.00 ^cd^	0.08 ± 0.01 ^d^	0.08 ± 0.01 ^cd^	0.08 ± 0.00 ^cd^
	10	0.09 ± 0.01 ^abc^	0.09 ± 0.01 ^cd^	0.09 ± 0.01 ^cd^	0.08 ± 0.01 ^cd^
	15	0.05 ± 0.01 ^e^	0.05 ± 0.01 ^e^	0.04 ± 0.00 ^e^	0.05 ± 0.01 ^e^
RP (eqv. mg mL^−1^ ascorbic acid per g of product)	0	0.30 ± 0.01 ^ef^	0.32 ± 0.01 ^cde^	0.32 ± 0.00 ^de^	0.33 ± 0.01 ^cd^
	5	0.33 ± 0.01 ^cd^	0.25 ± 0.03 ^g^	0.34 ± 0.01 ^bcd^	0.36 ± 0.01 ^a^
	10	0.34 ± 0.01 ^bcd^	0.32 ± 0.01 ^cd^	0.34 ± 0.01 ^abc^	0.36 ± 0.0 ^ab^
	15	0.23 ± 0.00 ^h^	0.25 ± 0.01 ^g^	0.28 ± 0.01 ^f^	0.25 ± 0.00 ^gh^

PP0—without sodium nitrite addition; PP50with sodium nitrite addition in the amount of 50 mg kg^−1^; PP100—with sodium nitrite addition in the amount of 100 mg kg^−1^; PP150—with sodium nitrite addition in the amount of 150 mg kg^−1^. Means with different lowercase letters (a–h) differ significantly (*p* < 0.05).

**Table 10:** Factor coordinates of the variables, based on correlations.

Variables	PC1	PC2	PC3
L*	0.172	−0.006	**−0.645**
a*	0.353	−0.357	**0.506**
b*	−0.118	0.037	**0.595**
N	−0.074	0.282	−0.068
TP	−0.243	**0.884**	0.203
HI	−0.243	**0.884**	0.203
a_w_	**−0.804**	−0.415	−0.087
pH	0.008	0.338	**−0.514**
TBARS	**0.710**	0.424	−0.181
POV	**−0.857**	0.293	0.096
TAC_ABTS	−0.290	**0.595**	−0.305
PEP_ABTS	**−0.920**	−0.008	−0.083
TAC_RP	**0.559**	−0.486	−0.135
PEP_RP	**−0.586**	**−0.615**	−0.143
PEP_DPPH	**−0.834**	−0.326	−0.083

Correlations in bold are higher than 0.500 and lower than −0.500. L*, a*, b*—color coordinates, N—nitrosylhemochrom content, TP—total pigments content, HI—heme iron content, TAC_ABTS—ABTS•^+^ radical scavenging activity of meat—water extract, TAC_RP—reducing power on meat—water extract, PEP_ABTS—ABTS^*+^ radical scavenging activity of extracted peptides, PEP_DPPH—DPPH radical scavenging activity of extracted peptides; PEP_RP—reducing power of extracted peptides.
